# Evaluation of Patterns of Liver Toxicity in Patients on Antiretroviral and Anti-Tuberculosis Drugs: A Prospective Four Arm Observational Study in Ethiopian Patients

**DOI:** 10.1371/journal.pone.0094271

**Published:** 2014-04-08

**Authors:** Getnet Yimer, Marcus Gry, Wondwossen Amogne, Eyasu Makonnen, Abiy Habtewold, Zelalem Petros, Getachew Aderaye, Ina Schuppe-Koistinen, Lars Lindquist, Eleni Aklillu

**Affiliations:** 1 Division of Clinical Pharmacology, Department of Laboratory Medicine, Karolinska University Hospital Huddinge C1:68, Karolinska Institutet, Stockholm, Sweden; 2 Department of Pharmacology, School of Medicine, College of Health Sciences, Addis Ababa University, Addis Ababa, Ethiopia; 3 Former AstraZeneca R&D, Global Safety Assessment, Molecular Toxicology, Södertälje, Sweden; 4 Department of Internal Medicine, School of Medicine, College of Health Sciences, Addis Ababa University, Addis Ababa, Ethiopia; 5 AstraZeneca Innovative Medicines Personalised Healthcare & Biomarkers, Science for Life Laboratory, Solna, Sweden; 6 Department of Medicine, Division of Infectious Diseases, Karolinska Institute at Karolinska University Hospital Huddinge I:73, Stockholm, Sweden; Vanderbilt University, United States of America

## Abstract

**Objectives:**

To evaluate the incidence, type, severity and predictors of antiretroviral and/or anti-tuberculosis drugs induced liver injury (DILI).

**Methods:**

A total of 1,060 treatment naive patients were prospectively enrolled into four treatment groups: HIV patients receiving efavirenz based HAART alone (Arm-1); TB-HIV co-infected patients with CD4≤200 cells/μL, receiving concomitant rifampicin based anti-TB and efavirenz based HAART (Arm-2); TB-HIV co-infected patients with CD4>200 cells/μL, receiving anti-TB alone (Arm-3); TB patients taking rifampicin based anti-TB alone (Arm-4). Liver enzyme levels were monitored at baseline, 1st, 2nd, 4th, 8th, 12th and 24th weeks during treatment. CD4 and HIV viral load was measured at baseline, 24th and 48th weeks. Data were analyzed using multivariate Cox Proportional Hazards Model.

**Results:**

A total of 159 patients (15%) developed DILI with severity grades 1, 2, 3 and 4 of 53.5%, 32.7%, 11.3% and 2.5% respectively. The incidence of cholestatic, hepatocellular or mixed pattern was 61%, 15% and 24%, respectively. Incidence of DILI was highest in Arm-2 (24.2%)>Arm-3 (10.8%)>Arm-1 (8.8%)>Arm-4 (2.9%). Concomitant anti-TB-HIV therapy increased the risk of DILI by 10-fold than anti-TB alone (p<0.0001). HIV co-infection increased the risk of anti-TB DILI by 4-fold (p = 0.004). HAART associated DILI was 3-fold higher than anti-TB alone, (p = 0.02). HAART was associated with cholestatic and grade 1 DILI whereas anti-TB therapy was associated with hepatocellular and grade ≥ 2. Treatment type, lower CD4, platelet, hemoglobin, higher serum AST and direct bilirubin levels at baseline were significant DILI predictors. There was no effect of DILI on immunologic recovery or virologic suppression rate of HAART.

**Conclusion:**

HAART associated DILI is mainly cholestatic and mild whereas hepatocellular or mixed pattern with high severity grade is more common in anti-tuberculosis DILI. TB-HIV co-infection, disease severity and concomitant treatment exacerbates the risk of DILI.

## Introduction

Antiretroviral and anti-tuberculosis chemotherapy associated drug induced liver injury (DILI) is a common and challenging adverse event causing adherence problem leading to hospitalization and life-threatening events [1]–[4]. DILI can be fatal if therapy is not interrupted on time, and the subsequent adherence problem may cause treatment failure and relapse or drug resistance [5]–[7]. Discontinuation of antiretroviral therapy in HIV infected individuals due to DILI is on rise reaching up to 32% [8]. About 8% to 23% of HIV-infected patients receiving highly active antiretroviral treatment (HAART) develop DILI and the pathogenic mechanisms are not fully understood [3], [9]. We recently reported the association of high efavirenz plasma concentration and CYP2B6*6 allele coding for slow efavirenz metabolizer phenotype with efavirenz based HAART associated DILI in TB-HIV patients [10]–[12]. A recent case report of efavirenz induced acute liver failure requiring liver transplantation in a slow drug metabolizer indicates fatal event in susceptible patients [13]. Consequently identification of the risk and prognostic factors is critical to identify patients at risk of developing DILI drugs for proper management.

All classes of antiretroviral drugs and some anti-TB drugs such as pyrazinamide, isoniazid and rifampicin are identified as potential cause of DILI [1], [3]. The type and incidence of DILI display wide differences between population and geographical location [14]–[16]. Severe DILI due to HAART is more frequent among Hispanics compared to other populations [14]. Anti-tuberculosis agents are the leading cause for DILI in India, in contrast to acetaminophen in the US and the UK [17]–[19]. The reported incidence of anti-TB therapy and/or HAART associated DILI within Africa varies greatly [10], [15], [16], [20]–[23]. Recent studies indicate association of pharmacogenetic variation with DILI [10], [11], [24]–[26]. Accordingly due to wide genetic heterogeneity in African populations, extrapolation of results from one population to another within the continent is challenging. Therefore more studies are urgently needed to explore the incidence, severity, type and predictors of liver injury associated with antiretroviral and anti-TB therapy for development of target oriented treatment guidelines in Sub-Saharan Africa, a continent highly affected by HIV/AIDS and tuberculosis.

Understanding the incidence, predictors and clinical pattern of antiretroviral and/or anti-TB drugs associated liver injury is hampered by differences in the study populations, definitions of DILI used, lack of standard reference for upper normal limits of aminotransferases and monitoring as well as reporting practices. To the best of our knowledge, there is no systematic prospective observational study that compared and contrasted the incidence, severity, predictors and clinical pattern of HAART and/or anti-TB DILI using the same case definition and study population thereby controlling the effect of genetic variation. In the present study we performed a prospective observational study to evaluate the incidence, severity, pattern and predictors of HAART and/or anti-TB DILI in a large well defined cohort, four arm parallel treatment groups using the DILI case definition set by international DILI expert working group [27]. Effect of disease type (HIV, TB, hepatitis virus B and C co-infection), type of treatment received (HAART, anti-TB and combination thereof), baseline and follow up biochemical parameters on DILI were investigated in HIV patients receiving efavirenz based HAART alone, HIV negative TB patients receiving anti-TB drugs alone, HIV-TB co-infected patients receiving anti-TB drugs alone, and HIV-TB co-infected patients receiving both anti-TB drugs and HAART. The result indicates Antiretroviral and anti-TB drugs are more associated with cholestatic and hepatocellular liver toxicity, respectively and TB-HIV patients receiving concomitant anti-TB-HIV therapy have the highest risk of developing severe DILI.

## Materials and Methods

### Study design and setting

This observational prospective cohort study of standard clinical care was conducted from June 2007 to June 2011. All consecutive ARV and anti-TB naïve individuals who came to four study sites: Kazanchis, Arada, Beletshachew Health Centers and Black Lion specialized referral and teaching University Hospital in Addis Ababa, Ethiopia were enrolled.

The study protocol was approved by the Regional Ethical Review Board in Stockholm at Karolinska Institutet, Sweden; Institutional Review Board at Faculty of Medicine, Addis Ababa University; The National Ethics Review Committee at the Ethiopian Science and technology Ministry in Ethiopia. After a detailed explanation of the study, both verbally and in writing, written informed consent was obtained from all study participants.

### Study population and treatment

ARV and anti-TB treatment naïve individuals were enrolled prospectively, in Addis Ababa, Ethiopia. The eligibility criterias were women and men, age ≥18 years, non-pregnant women, receiving no other known hepatotoxic drugs concurrently, except cotrimoxazole 960 mg per day, which was given for all HIV positive patients before enrolment and during the follow up period following the Ethiopian national treatment guideline for TB and HIV. A total of 1060 patients were enrolled in parallel and assigned into four different types of treatment groups (Arms) based on type of treatment to receive, as decided by physicians working at the study sites according to the WHO and Ethiopian national TB and HIV treatment guidelines during the study period [28]: HIV patients without TB confection receiving efavirenz based HAART only (Arm-1, n = 273); TB-HIV co-infected patients with CD4 count ≤200 cells/μL receiving rifampicin based short course anti-TB drugs together with efavirenz based HAART (Arm-2, n = 495); TB-HIV co-infected patients with CD4 count >200 cells/μL receiving anti-TB drugs alone (Arm-3, n = 83); TB patients without HIV confection receiving anti-TB drugs alone (Arm-4, n = 209). Treatment type, duration and dosing schedule as well as patient management was provided, by patients own physicians strictly following the WHO and national TB and HIV treatment guidelines considering the type of disease (TB, HIV, TB-HIV co-infection) and pretreatment CD4 cell count [28]. Being an observational study, no treatment modification was done for the study purpose and the study investigators had no influence on the physician's decisions regarding treatment choice or management of patients.

Treatment was initiated following the national treatment guideline of Ethiopia for TB and HIV. All TB patients received short-course anti-TB chemotherapy consisting of rifampicin, ethambutol, pyrazianmide and isoniazid for the first two months followed by isoniazid and rifampicin for the next four months. HIV patients in Arm-1 and Arm-2 treatment group received efavirenz 600 mg based highly active antiretroviral therapy (HAART) containing stavudine/lamivudine/efavirenz (D4T/3TC/EFV) or zidovudine/lamivudine/efavirenz (AZT/3TC/EFV) or tenofovir/lamivudine/efavirenz (TDF/3TC/EFV). None of the study participants received isoniazid prophylaxis and treatment for tuberculosis two years prior to study enrolment.

### Data collection and laboratory investigations

The socio-demographic characteristics, detailed history of present and past illnesses were recorded with findings from a general physical examination using a case record format and questioner prepared for the study. The study physicians performed clinical evaluations for any adverse events and monitoring of patients' progress at every clinical visit. Baseline laboratory investigations including differential blood counts, platelet count, CD4 count (for HIV positive patients), HIV RNA determination (for HIV positive patients), hepatitis B surface antigen, anti-hepatitis C antibody, serum albumin, creatinine, liver tests including; aspartate aminotransferase (AST), alanine aminotransferase (ALT), alkaline phosphatase (ALP), and direct and total bilirubin were done before treatment initiation. Monitoring of liver enzymes levels were performed at baseline and on the 1^st^, 2^nd^, 4^th^, 8^th^, 12^th^, 24^th^, and 48^th^ weeks after initiation of treatment. CD4 and HIV viral load was measured at week 24 and week 48. For the purpose of this study, censoring was done at 24 weeks, which is the time corresponding to completion of anti-TB therapy.

### Drug induced liver injury case definition, pattern and severity grade

We used the consensus criteria set by international DILI expert working group [27] for DILI case definition. The upper limit of normal (ULN) liver enzymes level for the study population was set based on the respective mean baseline value after exclusion of the 5% extreme values [29]. Accordingly the ULN of ALT for men and women were 33 U/L and 29 U/L respectively and for AST, ALP, total bilirubin and direct bilirubin were 41 U/L, 128 U/L, 1.0 mg/dL 0.3 mg/dL respectively. Patients were classified as DILI cases if any of the following criteria were met during any time point on therapy.

I. For patients whose baseline ALT or ALP were below the ULN:

≥ 5× ULN ALT, ≥ 2× ULN ALP or ≥ 3× ULN ALT and ≥ 2× ULN total bilirubin

II. For patients whose baseline ALT or ALP above the ULN:

≥ 5× ALT from baseline value, ≥2× ALP from baseline value or

≥3× ALT from baseline value and ≥2× ULN total bilirubin

Classification of DILI clinical presentation pattern was based on R-value defined as; 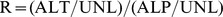

- Hepatocellular Pattern of DILI  =  R≥5
- Mixed Pattern of DILI  =  R>2 and <5
- Cholestatic pattern of DILI  =  R≤2

DILI severity grade was determined as defined by DILI Expert Working Group and AIDS Clinical Trials Group for patients having baseline ALT or ALP below ULN [27], [30] as described below.

- Grade 1: 1.25–2.5× ULN, or mild elevated ALT or ALP reaching criteria for DILI but bilirubin <2× ULN.
- Grade 2: 2.6–5.0× ULN, or moderate elevated ALT or ALP reaching criteria for DILI and total bilirubin ≥2× ULN.
- Grade 3: 5.1–10× ULN, or severe elevated ALT or ALP reaching criteria for DILI and total bilirubin ≥2× ULN and one of the following: INR ≥1.5, ascites, encephalopathy, disease duration <26 weeks, and absence of underlying cirrhosis, other organ failure considered due to DILI
- Grade 4: >10× ULN, or death or transplantation due to DILI

Abnormal levels of liver enzymes are common among persons infected with HIV and TB, and may be caused by multiple factors, including medication toxicity and coinfection with hepatitis C virus or hepatitis B virus. To avoid selection bias, for patients with elevated pretreatment serum liver enzyme levels higher than the ULN, the severity grading were classified based on changes relative to their respective baseline value rather than ULN as described previously [31].

- Grade 1: 1.25–2.5× baseline ALT or ALP
- Grade 2: 2.6–3.5× baseline ALT or ALP
- Grade 3: 3.6–5× baseline ALT or ALP
- Grade 4: >5× baseline ALT or ALP

*Management of drug toxicity and the stopping rule for treatment discontinuation was done according to the WHO Guiding principles for the management of ARV drug toxicity based on the severity grade* [28].

### Data analysis

Proportions (%) and median (interquartile range at 25th and 75th, IQR) were used to describe baseline socio demographic, clinical and laboratory parameters. Chi square and fishers exact tests were used to compare proportion between categorical variables using bonferroni adjustment for multiple comparisons. The data was right censored for the patients that did not fulfill the criteria's for drug induced liver injury. The variables (treatment group, BMI, type of HAART received, and baseline value for HBsAg, HCsAg, CD4 count, log HIV viral load, creatinine, urea, albumin, Log HIVRNA and neutrophil, platelet, AST, ALT, ALP, total and direct bilirubin) were used in a Univariate Cox proportional hazards model analysis to identify potential independent risk factors for DILI. The Efron approximation was used for ties in the time point of DILI onset. Parameters with p value <0.1 in the univariate analysis were incorporated in a multivariate cox regression analysis. In the multivariate Cox model, subgroup interaction terms were removed by a backward stepwise procedure. The data were analyzed using SPSS Statistics (IBM Corporation, Somers, NY) software, version 22.0. P value <0.05 was considered statistically significant.

## Results

A total of 1060 treatment naïve TB and/or HIV infected women (54.9%) and men (45.1%) patients were enrolled prospectively and assigned into four parallel treatment groups. Patients were followed for up to 48 weeks while on therapy for the development of clinical and biochemical DILI. Out of 1060 patients, 996 patients completed the study by the time of censoring at week-48. HIV patients in Arm-1 and Arm-2 received HAART regimen consisting of either AZT/3TC/EFV or D4T/3TC/EFV or TDF/3TC/EFV. All TB patients received short course rifampicin based anti-TB therapy following the national treatment guideline of Ethiopia during the study period. Baseline socio demographic and biochemical parameters for the study participants stratified by treatment group is presented in Table 1.

**Table 1:** Socio demographic, type of HAART, baseline clinical and laboratory characteristics of study participants stratified by treatment groups.

Parameters		Treatment group (Arm, n = 1060)			
		Arm1 (n = 273)	Arm2 (n = 495)	Arm3 (n = 83)	Arm4 (n = 209)
Sex	Female	200 (73.3%)	243 (49.1%)	39 (47.0%)	100 (47.8%)
	Male	73 (26.7%)	252 (50.9%)	44 (53.0%)	109(52.2%)
Education	Illiterate	48 (17.5%)	66 (13.4%)	8 (9.9%)	29 (14.1%)
	Primary	116 (42.6%)	202 (40.8%)	34 (40.8%)	80 (38.4%)
	Secondary	97 (35.7%)	190 (38.4%)	32 (38.0%)	72 (34.3%)
	Tertiary	11 (4.2%)	36 (7.3%)	9 (11.3%)	27 (13.1%)
HIV Stage	Stage 1	4 (1.5%)	1 (0.2%)	0	
	Stage 2	21 (7.7%)	4 (0.8%)	5 (6.0%)	
	Stage 3	108 (39.6%)4	337 (68.1%)	49 (59.0%)	
	Stage 4	140 (51.3%)	158 (33.1%)	29 (34.9%)	
Type of HAART	d4T/3TC/EFV	151 (55.3%)	158 (33.1%)		
	TDF/3TC/EFV	12 (4.4%)	182 (38.1%)		
	ZDV/3TC/EFV	110 (40.3%)	138 (28.9%)		
Hepatitis B surface antigen	negative	258 (94.5%)	452 (91.5%)	78 (95.1%)	205 (98.1%)
	positive	15 (5.5%)	42 (8.5%)	4 (4.9%)	4 (1.9%)
Hepatitis C virus antibody	negative	267 (97.8%)	484 (98.3%)	77 (93.9%)	205 (98.1%)
	positive	6(2.2%)	9(1.8%)	5 (6.1%)	4 (1.9%)
**Median (IQR)**					
Age		34 (28–40)	35 (30–42)	33 (27–41)	28 (23–40)
BMI		19.5 (17.8–21.9)	18.662 (16.8–20.3)	18.6 (17.4–20.6)	19.3 (17.5–21.5)
Karnofsky score		100 (90–100)	90 (80–100)	100 (90–100)	
Hemoglobin at baseline		12.8 (11–14)	11 (10–13)	12 (11–14)	13 (12–14)
WBC at baseline		4 (4–6)	6 (4–7.7)	6 (5–8)	7 (6–9)
Neutrophil at baseline		56 (47–65)	69 (60–77)	59 (49–68)	68 (60–75)
Platelet at baseline		240 (171–302)	296 (223–376)	247 (165–343)	387 (292–494)
AST at baseline		33 (27–44)	43 (30–74)	40 (31–62)	30 (24–41)
ALT at baseline		28 (21–39)	29 (20–42)	28 (24–39)	27 (21–39)
ALP at baseline		109 (88–129)	121 (91–174)	126 (110–165)	111 (98–138)
Urea at baseline		24 (19–30)	25 (20–32)	26 (20–35)	25 (21–31)
Creatinine at baseline		1 (1-1)	1 (1-1)	1 (1-1)	1 (1-1)
CD4 at baseline		104 (56–155)	79 (45–129)	279 (223–349)	
Log HIV viral load at baseline		5.21 (4.61–5.67)	5.09 (4.55–5.53)		

Arm1  =  HIV patients treated with efavirenz based HAART only.

Arm2  =  TB-HIV patients with baseline CD4<200 cells/μL, treated with efavirenz based HAART and rifampicin based anti-tuberculosis drugs.

Arm3  =  TB-HIV patients with baseline CD4>200 cells/μL, treated with rifampicin based anti-tuberculosis drugs only,

Arm4  =  TB patients treated with rifampicin based anti-tuberculosis drugs only.

### Incidence of DILI

**Figure 1:**
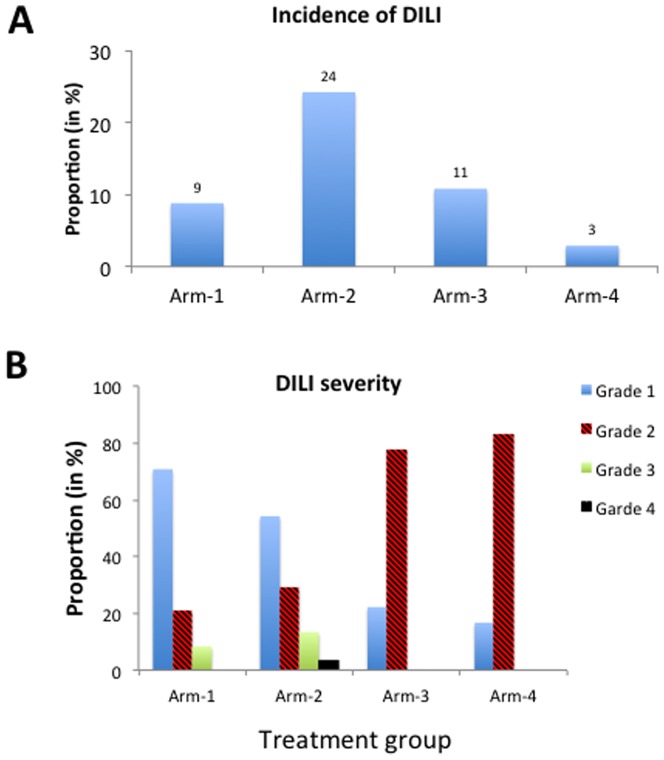
Comparison of incidence (Figure 1A) and severity grade of DILI in study participants stratified by treatment groups: HIV patients without TB co-infection (Arm-1) treated with efavirenz based HAART alone, TB-HIV co-infected patients with CD4 count ≤ 200 cells/μL treated with concomitant anti-TB and HAART therapy (Arm-2), TB-HIV co-infected patients with CD4 count >200 cells/μL treated with anti-TB therapy alone (Arm-3) and TB patients without HIV co-infection treated anti-TB therapy alone (Arm-4). Figure 1B indicates severity grade distribution among the total 159 DILI cases.

Incidence and severity of DILI stratified by treatment group is presented in Figure 1. Over all, out of the 1060 study participants, 159 patients (15%) developed DILI. Development of DILI was significantly associated with the disease status and type of treatment received (p<0.00001). Incidence of DILI was significantly higher in Arm-2 (n = 120, 24.2%) followed by Arm-3 (n = 9, 10.8%), then Arm-1 (n = 24, 8.8%) and last Arm-4 (n = 6, 2.9%). TB HIV co-infection and concomitant anti-TB-HIV therapy increased the risk of DILI by 10-fold than anti-TB therapy alone (Arm-2 vs. Arm-4, p<0.00001, OR = 10.83, 95%CI: 4.68 to 25.02). HIV co-infection increased the risk of anti-TB therapy DILI by 4-fold (Arm-3 vs. Arm-4, p<0.01, OR = 4.11, 95%CI: 1.45 to11.96). Incidence of DILI due to HAART alone was 3-fold higher than anti-TB therapy alone, (Arm-1 vs. Arm-4, p<0.02, OR = 3.21, 95% CI: 1.31 to 8.13).

### DILI severity grade

Among the total of 159 patients who developed DILI, the overall incidence of severity grades 1, 2, 3 and 4 DILI was 53.5% (n = 85), 32.7% (n = 52), 11.3% (n = 18) and 2.5% (n = 4), respectively (Table 2). The severity grade of DILI also varied significantly between the different treatment groups (Figure 1B). In HIV only patients treated with ARV alone (Arm-1), the incidence of grades 1, 2, and 3 DILI was 71% (n = 17), 21% (n = 5), 8% (n = 2), respectively and none of the patients had grade 4. On the other hand among TB-HIV patients treated with both ARV and anti-TB drugs (Arm-2), the incidence of grades 1, 2, 3 and 4 DILI was 54.2% (n = 65), 29.2% (n = 35), 13.3% (n = 16) and 3.3% (n = 4), respectively. Grade 2 DILI was more common in patients treated with anti TB drugs only (79% in Arm-3 and 83% in Arm-4) than grade 1 (22% in Arm-3 and 17% in Arm-4). None of the patients treated with anti TB drugs only (Arm-3 and Arm-4) had grade 3 or 4 severity.

**Table 2:** Comparison on incidence, type and severity grade of antiretroviral and/or antituberculosis DILI stratified by type of disease and treatment groups.

		Treatment group (n = 1060)				Total	P value
			
		Arm1 (n = 273)	Arm2 (n = 495)	Arm3 (n = 83)	Arm4 (n = 209)		
Incidence of DILI		24 (8.8%)	120 (24.2%)	9 (10.8%)	6 (2.9%)	15.0%	<0.0001
							
Types of DILI	Cholestatic	17 (70.8%)	70 (58.3%)	6 (66.7%)	4 (66.7%)	61.0%	<0.0001
	Hepatocellular	3 (12.5%)	18 (15.0%)	2 (22.2%)	1 (16.7%)	15.0%	
	Mixed	4 (16.7%)	32 (26.7%)	1 (11.1%)	1 (16.7%)	24.0%	
DILI severity grade	Grade 1	17 (70.8%)	65 (54.2%)	2 (22.2%)	1 (16.7%)	53.5%	<0.0001
	Grade 2	5 (20.8%)	35 (29.2%)	7 (77.8%)	5 (83.3%)	32.7%	
	Grade 3	2 (8.3%)	16 (13.3%)	0	0	11.3%	
	Grade 4	0	4 (3.3%)	0	0	2.5%	

Arm1  =  HIV patients treated with efavirenz based HAART only.

Arm2  =  TB-HIV patients with baseline CD4<200 cells/μL, treated with efavirenz based HAART and rifampicin based anti-tuberculosis drugs.

Arm3  =  TB-HIV patients with baseline CD4>200 cells/μL, treated with rifampicin based anti-tuberculosis drugs only,

Arm4  =  TB patients treated with rifampicin based anti-tuberculosis drugs only.

### Types of antiretroviral and/or antituberculosis drugs induced liver injuries

**Figure 2:**
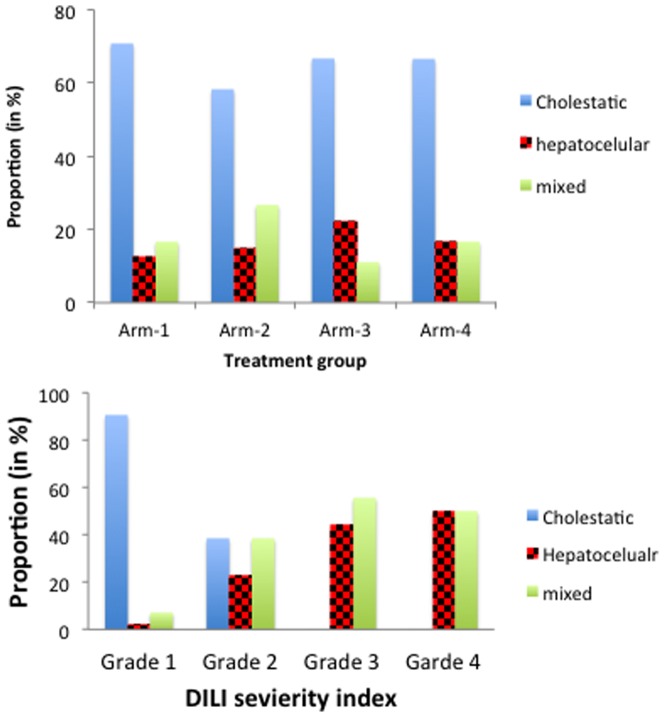
Distribution of the different types of liver injuries (hepatocellular, cholestatic and mixed type) that were observed in each treatment group (Figure 2A) and stratified by severity grade (Figure 2B): HIV patients without TB co-infection (Arm-1) treated with efavirenz based HAART alone, TB-HIV co-infected patients with CD4 count ≤ 200 cells/μL treated with concomitant anti-TB and HAART therapy (Arm-2), TB-HIV co-infected patients with CD4 count >200 cells/μL treated with anti-TB therapy alone (Arm-3) and TB patients without HIV co-infection treated anti-TB therapy alone (Arm-4).

Among the total of 159 patients who developed DILI, the overall incidence of cholestatic, hepatocellular and mixed pattern was 61% (n = 97), 15% (n = 24) and 24% (n = 38), respectively (Table 2). Incidence of cholestatic DILI in Arm-1, Arm-2, Arm-3 and Arm-4 was 71%(n = 17), 58% (n = 70), 67% (n = 6) and 67% (n = 4), respectively (Figure 2A). Incidence of mixed cholestatic and hepatocellular DILI in Arm-1, Arm-2, Arm-3 and Arm-4 was 17% (n = 4), 27% (n = 32), 11% (n = 1) and 17% (n = 1), respectively. Hepatocellular DILI was highest among patients treated with anti TB drug alone (22% in Arm3 n = 2, 17% in Arm-4 n = 1) or with HAART (15% in Arm-2, n = 18) than patients treated with HAART alone (12.5% in Arm-1, n = 3).

There was a significant interaction between DILI pattern, severity grade and type of treatment received. Irrespective of treatment group, none of the patients presenting cholestatic DILI had grade 3 or 4 (Figure 2B). In contrast grade 3 and 4 was common among patients presenting hepatocellular (33% and 8.3%, respectively) or mixed (26% and 5.3%, respectively) pattern.

A total of 22 patients developed DILI with severity grade 3 or above and all of them were TB-HIV coinfected patients receiving concomitant antiretroviral and antituberculosis therapy, except two who were HIV only infected patients receiving HAART (Table 2). The clinical and biochemical progress of DILI were closely monitored in patients who developed severity grade 3 (n = 18) and grade 4 (n = 4). None of the patients who had grade 3 DILI discontinued their therapy, as the level of liver enzymes dropped gradually and they were not clinically symptomatic. However, four TB-HIV coinfected patients who developed DILI severity grade 4 discontinued both HAART and anti TB treatment with gradual successful re-introduction. As indicated in Figure 2, none of these four patients who developed severity grade 4 DILI had cholestatic DILI, instead hepatocellular and mixed type of DILI constituted 50% each. After all the signs and symptoms of DILI were relived and the liver enzyme level dropped down to the normal range, patients were reinitiated on their anti-tuberculosis therapy according to the desensitization protocol described previously [22] followed by HAART.

### Predictors of DILI

**Figure 3:**
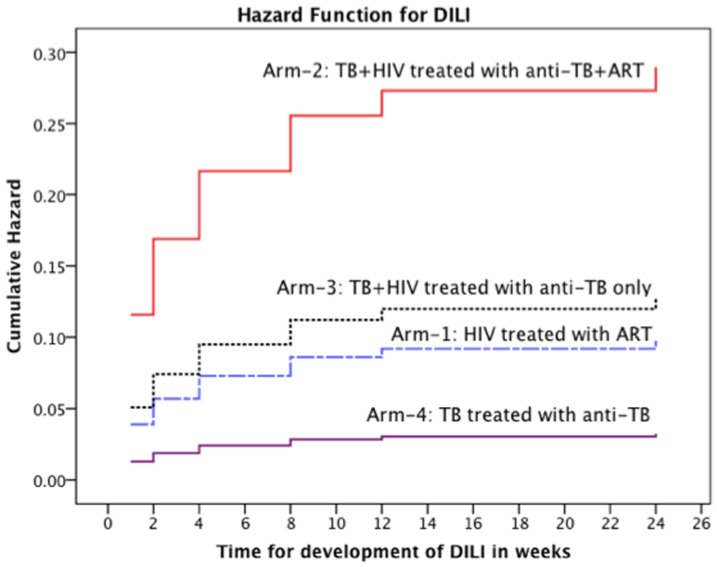
Kaplan–Meier curves to estimate cumulative hazard for the development of DILI stratified by type of treatment groups: HIV patients without TB co-infection (Arm-1) treated with efavirenz based HAART alone, TB-HIV co-infected patients with CD4 count ≤ 200 cells/μL treated with concomitant anti-TB and HAART therapy (Arm-2), TB-HIV co-infected patients with CD4 count >200 cells/μL treated with anti-TB therapy alone (Arm-3) and TB patients without HIV co-infection treated anti-TB therapy alone (Arm-4).

The median time for DILI onset among Arm-1, Arm-2, Arm-3 and Arm-4 patients was 4, 1, 2 and 6 weeks, respectively. Univariate and multivariate Cox Proportional hazard regression analyses of possible factors associated with DILI is listed in Table 3. Kaplan–Meier estimates were calculated to adjust for the censoring of data up to week 48. Cox regression hazard ratio stratified by treatment group is presented in Figure 3. The univariate analysis indicated treatment group, lower BMI, Karnofsky scores, hemoglobin, neutrophil, platelet, albumin and high AST, ALT, ALP, direct bilirubin and HIV viral load at baseline as independent predictors. All variables in the Univariate Cox Proportions hazard regression analysis with a p<0.1 were entered into the multivariate cox regression model. Backward conditional multivariate regression analysis retained treatment group (Arm), low hemoglobin, high AST and direct bilirubin at baseline as significant predictors of DILI in the model.

**Table 3:** Univariate and Multivariate Cox proportional regression analysis to show the risk factors for developing DILI. HR  =  hazard ratio.

Variable		Univariate analysis		Multivariate analysis	
		HR (95% CI)	p-value	HR (95% CI)	p-value
WHO clinical stage (Ref stage 4)			0.37		
	Stage 1	0.31 (0.03–3.45)	0.34		
	Stage 2	0.95 (0.13–6.82)	0.96		
	Stage 3	0.80 (0.11–5.84)	0.83		
Arm: Diseases and treatment group			<0.0001	0.62 (0.49–0.79)	<0.0001
(Ref: Arm4, TB patients treated with anti-TB therapy alone)	Arm1: HIV patients treated with HAART	3.02 (1.23–7.40)	0.016		
	Arm2: TB-HIV patients treated HAART + anti TB	8.95 (3.94–20.34)	<0.0001		
	Arm3: TB-HIV patients treated with anti TB	3.93 (1.40–11.05)	0.009		
Age		1.00 (0.98–1.07)	0.79		
Male sex		0.59 (0.76–1.62)	0.59		
Body Mass Index		0.93 (0.88–0.98)	0.013	1.01 (0.94–1.07)	0.87
Karnofsky Scores (ref – Karnofsky score <80%)		1.68 (1.21–2.32)	0.002	0.99 (0.98–1.01)	0.62
Type of HAART received		1.06 (0.87–1.28)	0.57		
Baseline Neutrophil		1.019 (1.00–1.03)	0.003	1.01 (0.99–1.02)	0.46
Baseline hemoglobin		0.82 (0.77–0.88)	<0.0001	0.92 (0.86–0.99)	0.04
Baseline WBC		0.98 (0.92–1.041)	0.51		
Baseline platelet		0.99 (0.98–0.99)	0.002	0.99 (0.99–1.00)	0.03
Baseline AST		1.00 (1.00–1.00)	<0.0001	1.01 (1.00–1.01)	0.001
Baseline ALT		1.00 (1.00–1.01)	0.026	1.00 (0.99–1.01)	0.62
Baseline ALP		1.00 (1.00–1.00)	<0.001	1 (0.999–1.002)	0.52
Baseline Bilirubin (Total)		0.98 (0.87–1.10)	0.78		
Baseline Bilirubin (Direct)		1.72 (1.26–2.33)	0.001	1.49 (1.09–2.04)	0.01
Baseline Albumin		0.572 (0.47–0.70)	<0.0001	0.92 (0.74–1.14)	0.46
Baseline Creatinine		1.01 (0.73–1.40)	0.94		
Baseline urea		1.00 (0.99–1.019)	0.89		
Hepatitis B positive (ref – HbsAg negative)		1.13 (0.61–2.08)	0.69		
Hepatitis C positive (ref – HCV Ab negative)		1.19 (0.44–3.21)	0.73		
Baseline CD4 cell count		0.99 (0.99–1.00)	0.003	0.99 (0.99–1.00)	0.03
Log baseline HIV RNA Viral load		1.26 (1.014–1.58)	0.04	0.93 (0.86–1.00)	0.41

### Effect of DILI on HAART outcome

Among HIV patients treated with HAART (Arm-1 and Arm-2), there was no significant effect of DILI on the percent change in CD4 cell count from baseline or proportion patients with detectable viral load (>50 copies/mL) or HIV viral load >200 copies/mL by week 24 or 48 weeks of HAART (p>0.05).

## Discussion

We performed a prospective parallel assignment observational study to evaluate the incidence, type, severity and predisposing risk factors of DILI as well as effect of DILI on HAART efficacy outcome in a large well defined TB and/or HIV patient cohort receiving either anti-TB therapy alone, HAART alone or concomitant anti-TB and antiretroviral therapy. Our major finding includes i) ARV induced liver injury is associated mainly with cholestatic and mild injury (grade-1). In contrast hepatocellular or mixed type of liver injury pattern with severity grade ≥ 2 is more common among TB patients receiving anti-TB drugs with or without HAART ii) TB-HIV co-infection and disease severity exacerbates the risk of DILI. iii) although lower BMI, higher HIV viral load, and abnormal liver enzymes level at baseline were implicated as independent risk factors in the univariate model, a multivariate analysis indicated disease status and type of treatment received, low hemoglobin, CD4 cell count, high AST and direct bilirubin at baseline as predictors of DILI. To the best of our knowledge this is the first systematic study to investigate the incidence, type, severity and predictors of DILI stratified by disease status and type of treatment received in the same population there by controlling for effect of genetic predisposition to DILI from Africa.

Evaluations of the incidence, type of injury, severity grade and identification of prognostic markers for DILI requires parallel and equivalent assessment of the aminotransferases and use of the same DILI phenotype classification [32]. Inconsistent definitions and terminology related to the clinical phenotypes of DILI, lack of a reference upper limit of normal (ULN) for serum aminotransferase levels and inter-laboratory variability in the expression ULN and use of ethnically diverse study population is a challenge for cross-study comparisons of DILI association studies [27], [29]. The use of same DILI case definition across the different parallel treatment groups in ethnically the same study population makes our study ideal to compare the incidence, pattern, severity grade and risk factors of ARV and/or anti-TB DILI between HIV, TB and TB-HIV infected patients.

The incidence of hepatocellular, cholestatic or mixed type of DILI stratified by severity grading and by treatment groups is presented in Table 2 and Figure 2. Hepatocellular hepatotoxicity is generally associated with marked elevation in aminotransferase levels (ALT, AST, or both), which may be followed by hyperbilirubinemia in severe cases. Cholestatic hepatotoxicity is generally characterized by marked elevation of serum alkaline phosphatase levels and development of pruritus and jaundice. In mixed type of clinical syndromes, neither aminotransferase nor alkaline phosphatase elevations are clearly predominant. In our study population, cholestasis pattern of liver injury accounted >50% of the DILI cases being highest in HIV patients treated with efavirenz based HAART alone (71%). Drugs that are excreted by the liver into bile and drugs that inhibit bile acid transport processes are prime suspects to elicit cholestatic liver injury. Several antiretroviral including efavirenz inhibit bile acid transport in human and rat hepatocytes [33]. A study in healthy volunteers reported decreased unconjugated and conjugated bilirubin by efavirenz possibly via induction of UGT1A1 and bile efflux transporters respectively [34] and this may result in unanticipated drug interactions, altering excretion of other concomitant drugs used in combination therapy. The observed cholestasis liver injury was more of transient and mild. The low severity grade of efavirenz based HAART is accordance with other reports [35]. Though mild in most patients, efavirenz based HAART induced liver injury can be life threatening in susceptible individuals [13]. In line with this a total of 29% of the DILI cases had hepatocellular or mixed hepatocellular with severity grade 2 or 3. Thus close monitoring of liver enzyme particularly in mono HIV infected patients presenting particularly hepatocellular pattern soon after initiation of HAART is crucial for proper management. On the other hand, hepatocellular or mixed hepatocellular and cholestasis type liver injury was more common in TB patients treated with anti-TB along with (40% in Arm2) or with out HAART (33% in Arm 3 and 4).

About one forth (24%) of the TB-HIV co-infected patients treated with both anti-TB drugs and HAART concomitantly developed DILI and 46% of them had severity grade ≥ 2. The lowest DILI incidence was recorded among HIV negative TB patients who received anti-TB drugs alone (2.8%) but the majority 5 out of 6 (83%) had grade-2 liver injury. The observed incidence of DILI among TB-HIV patients treated with concomitant ARV and anti-TB drugs (24%) was much higher than the sum of the incidences in HIV patients treated with ARV alone (Arm-1, 8.1%) and TB-HIV patients treated with anti TB alone (10.8%). Accordingly DILI in TB-HIV co-infected patients receiving concomitant HAART and anti-TB therapy is rather a toxic synergism than a simple additive effect. TB and HIV disease co-morbidity might have exacerbated the combined toxicity of anti TB drugs and HAART. On the other hand complex drug interactions between ARV and anti-TB drugs involving enzyme induction and inhibition may accentuate the overlapping toxicities. We recently reported the association of higher plasma level of efavirenz among patients who developed DILI during HAART [10]. Rifampicin, a potent inducer of cytochrome P450 enzymes and drug transporter is reported to decrease the serum level of efavirenz. Furthermore efavirenz undergoes auto induction lowering its own plasma exposure overtime [36] though this phenomenon was not observed in Ethiopian HIV patients [37]. Consequently lower incidence of efavirenz associated liver injury is expected among patients on concomitant efavirenz–rifampicin therapy. However recent studies in sub-Saharan African populations indicate rifampicin co-administration has no significant effect or even paradoxically increases efavirenz plasma concentration [38], [39]. Hence overlapping toxicities between anti-TB and ARV drugs contributes for the higher incidence of DILI in co-infected patients.

The median time for the development of DILI was much shorter in TB-HIV co-infected patients treated with ARV and/or anti TB drugs, presenting with in the first 2 weeks of therapy initiation, than HIV or TB alone infected patients receiving ARV or anti-TB drugs, respectively. Accordingly close monitoring of liver enzymes soon after therapy initiation is recommended for patients with dual infection of TB and HIV. Not only the incidence of DILI is high in TB-HIV patients treated with concomitant TB-HIV therapy, but also the severity grade is much higher compared to patients treated with ARV only or anti-TB drug only. Grade 3 or 4 toxicity was more common among TB-HIV patients treated with concomitant HAART plus anti-TB drugs (16%) or ART alone (8.3%). In deed grade-4 DILI (3.3%) was observed only in TB-HIV co-infected patients treated with concomitant anti-TB and HAART. In all HIV patients, the surrogate markers for HIV diseases severity (lower CD4 count and higher HIV RNA load at baseline) were significant risk predictors of DILI. In the present study, we found no significant effect of DILI on immunological recovery and virology treatment outcome of HAART similar to the recent study from Tanzania [12].

Considering TB patients who received anti-TB chemotherapy alone, the DILI incidence was significantly higher in HIV co-infected patients (Arm-3) than those without HIV co-infection (Arm-4), and the DILI event occurs much earlier during therapy. Apart from differences in the disease status, all HIV positive TB patients were also initiated with cotrimoxazole, which might have exacerbated the liver injury. However HIV only patients in Arm1 also received cotrimoxazole and yet the DILI incidence is in Arm1 is lower than Arm3. Hence the most likely cause is TB-HIV co-infection itself, which might contribute for increased risk of steatohepatitis and advanced fibrosis [40], [41].

In general concomitant HAART and anti-TB therapy increases the risk for DIIL 10 times more than anti-TB alone. Though mild, HAART alone increases the risk for DIIL 3 times higher than anti-TB alone in Ethiopian HIV patients. Identification of high-risk group of patients for HAART and anti-TB associated DILI and its clinical presentation pattern helps policy makers to establish target oriented treatment guidelines and polices in fighting against HIV/AIDS and tuberculosis in resource-limited countries. A recent study from Uganda reported low incidence of severe hepatotoxicity within three months of first-line HAART and concluded that routine measurement of transaminases may not be necessary in all patients initiating HAART in resource limited settings [15]. In contrast, our study demonstrates that HAART associated DILI is frequent in Ethiopian HIV patients requiring regular monitoring of liver enzyme and clinician's attention for proper DILI management. Accordingly caution needs to be applied in direct extrapolation of findings from one study population to another.

In conclusion, type of treatment received, diseases status and progression influence the incidence, type and severity of DILI. Our results confirm TB-HIV co-infected patients receiving concomitant TB-HIV therapy are at a higher risk to develop severe DILI than HIV patients receiving HAART alone or TB patients receiving anti TB alone. HAART induced DILI is mainly associated with cholestatic pattern with mild severity grade. Though mild in most patients, antiretroviral induced liver injury can be severe in susceptible individuals. Hence close monitoring of patients with antiretroviral induced liver injury presenting hepatocellular type is recommended for proper management. Hepatocellular or mixed pattern with relatively higher severity grade is common with anti-TB induced liver toxicity. Regardless of treatment type or disease status, patients with hepatocellular DILI are at a higher risk of developing severe DILI. HAART and anti-TB DILI is frequent and a major concern among Ethiopian TB-HIV patients and regular monitoring of liver enzymes during early therapy is recommended for proper identification and management of DILI.
